# Antimicrobial Efficacy of *Teucrium polium* L. Extracts for Dental Caries: Green Extraction Techniques and Bioactive Compounds

**DOI:** 10.1002/fsn3.4718

**Published:** 2025-01-07

**Authors:** Kamand Javadpour, Hajar Shekarchizadeh, Mohammad Goli, Helena Moradiyan Tehrani

**Affiliations:** ^1^ Community Health Research Center, Department of Community Oral Health, School of Dentistry, Isfahan (Khorasgan) Branch Islamic Azad University Isfahan Iran; ^2^ Department of Food Science and Technology, Damghan Branch Islamic Azad University Isfahan Iran; ^3^ Department of Food Science and Technology, Laser and Biophotonics in Biotechnologies Research Center, Isfahan (Khorasgan) Branch Islamic Azad University Semnan Iran

**Keywords:** antibacterial aqueous‐ethanoic extract, dental caries, disc inhibition zone, minimum inhibitor concentration, *Teucrium polium* L., well inhibition zone

## Abstract

Dental caries is a highly prevalent chronic condition globally. In recent years, scientists have turned to natural compounds such as plant extracts as an alternative to address concerns related to biofilm‐mediated disease transmission, increasing bacterial resistance, and the adverse impacts of antibiotics. Consequently, this study investigated the antimicrobial properties of ethanolic, hydroethanolic, and aqueous extracts of *Teucrium polium* L. (*T. polium*), which belongs to the *Lamiaceae* family, at different concentrations (0.1, 1, 10, 100, 500, 1000 ppm) against seven bacteria commonly associated with dental decay. The hydroethanolic extract demonstrated the highest efficacy against *S. mutans* (minimum inhibitory concentration (MIC) = 1.24 mg/mL), while the ethanolic extract exhibited the most potent activity against *S. sanguinis* (MIC = 1.55 mg/mL). For *S. sobrinus* , the ethanolic extract was the most effective (MIC = 1.52 mg/mL), whereas the hydromethanolic extract displayed the highest efficacy against *S. salivarius* (MIC = 1.52 mg/mL). *S. aureus* was most susceptible to the ethanolic extract (MIC = 1.9 mg/mL), whereas the aqueous extract demonstrated the strongest antimicrobial effect against *S. epidermidis* (MIC = 2.03 mg/mL). Finally, the ethanolic extract exhibited the maximum efficacy against *L. fermentum* (MIC = 1.36 mg/mL). Overall, the ethanolic extract demonstrated the highest efficacy against all tested bacteria, followed by the hydroethanolic extract, while the aqueous extract showed comparatively lower effectiveness. Therefore, depending on the specific target bacteria, it is suggested to combine the antibacterial extract of *T. polium* with the most effective solvent to effectively combat the bacteria responsible for dental decay. The study found that mouthwashes containing ethanolic and hydroethanolic extracts, at a concentration of 2.44 mg/L, effectively inhibited the growth of all oral bacteria contributing to dental caries. Future research should explore *T. polium* extracts' mechanisms of action against oral pathogens, their practical applications, and their efficacy against conventional treatments, paving the way for innovative dental therapies.

## Graphical abstract

*Teucrium polium* extracts effectively inhibit oral bacteria causing dental caries, with alcoholic extracts outperforming aqueous extracts. Mouthwashes containing *T. polium*'*s* effective component prevent tooth decay.

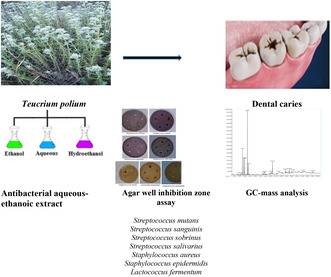

**Abbreviations**
EOs: Essential oils
GC–MS: Gas chromatography–mass spectroscopy

*L. fermentum*

: *Lactococcus fermentum*
MIC: Minimum inhibitory concentration
OD: Optical density

*S. aureus*

:
*Staphylococcus aureus*

*S. epidermidis*

:
*Staphylococcus epidermidis*

*S. mutans*

:
*Streptococcus mutans*

*S. salivarius*

:
*Streptococcus salivarius*

*S. sanguinis*

:
*Streptococcus sanguinis*

*S. sobrinus*

:
*Streptococcus sobrinus*

*T. polium*
: *Teucrium polium* L.
ZOI: Zone of inhibition

## 1 Introduction

Dental caries, often known as tooth decay or cavities, is one of today's most common and pervasive chronic diseases, as well as one of the most avoidable issues in dental care (Uadav and Prakash 2017). Dental caries often develops when the tooth's hard tissue (enamel and eventually dentin) is gradually damaged by acid production from decay‐causing bacteria. As the enamel deteriorates, the tooth loses its ability to naturally repair the calcium and phosphate structures of the teeth via saliva qualities, and acid eventually penetrates the tooth and destroys it from the inside out (Bin et al. 2020). Dental biofilm is a term used to describe the polymicrobial communities formed by the oral microbiota on the surface of teeth. One of the key factors contributing to dental caries is the buildup of bacterial biofilm. The majority of microorganisms that penetrate the oral cavity have the potential to be harmful (Marsh and Zaura 2017). They integrate themselves into the native flora and change the biofilm ecosystem, causing the host to mount an immunological and inflammatory response. Therefore, it can be argued that an ecological imbalance in the mouth's natural flora is one of the causes of caries; restoring the oral flora is one of the most efficient ways to prevent dental caries (Chen et al. 2020).

The majority of the diagnostic and treatment approaches are directed at the sugar‐fermenting acidogenic species of *S. mutans* , which has been the focus of dental caries research for several decades. Although *S. mutans* only makes up a minor portion of the bacterial population, new DNA‐ and RNA‐based investigations of caries have revealed a very complex ecosystem (Bodiba et al. 2018). Although it is not the cause of caries, *Lactobacillus* is thought to be the second species in the mouth to contribute to its development. There is a lot of evidence that *L. fermentum* is carious. Additionally, *S. aureus* , *S. epidermidis* , and *S. sobrinus* can all contribute to the development of caries. However, studies have shown that an increase in the bacteria *S. sanguinis* and *S. salivarius* can diminish the bacteria *S. mutans* and help to prevent tooth decay (Pitts et al. 2017).

Given the significance of biofilm in the emergence of illnesses, the propagation of microbial resistance, and the negative effects of antibiotics, there is a global movement toward the use of plant extracts as caries prevention and oral hygiene (dos Santos Cardoso et al. 2021). A common medicinal plant in Southwestern Asia is *Teucrium polium* L. (*T. polium*), which belongs to the Lamiaceae family. *T. polium* has historically been used to treat a variety of pathological illnesses, including rheumatism, diabetes, and digestive disorders. *T. polium* includes several bioactives, such as, saponin, flavonoid, glycoside‐alpha, tannin, terpenoid, diterpenoid, linalool, sterol, leucoanthocyanin, betacaryophyllene, hemolen, caryophyllene oxide, asparagine, and ditrin (Khazaei et al. 2018). This plant's antibacterial and antifungal properties have also been demonstrated in numerous research. Additionally, the previous researchers looked into how a mouthwash containing the plant *T. polium* affects the population of salivary *S. mutans* . They claimed that the colonization of *S. mutans* in human saliva was greatly inhibited by the aqueous extract of *T. polium* (Khoramian Tusi et al. 2020).

The goal of this study was to ascertain the antimicrobial impact of aqueous, ethanolic, and hydroethanolic extracts of *T. polium* on both the beneficial bacteria in the oral biofilm and the caries‐causing bacteria. This was done because, despite numerous studies on the properties of *T. polium*, none have been done on the effect of different *T. polium* extracts on tooth decay.

## 2 Materials and Methods

### 2.1 Materials

The plant used in this study was gathered in June 2021 from the hills surrounding Khatam city in Yazd region, Iran, at the peak of its flowering cycle. The plant used in this study, *T. polium*, was collected in June 2021 from the hills surrounding Khatam city in the Yazd region of Iran, at the peak of its flowering cycle. The Yazd province's research and training center for agriculture and natural resources, with the associated herbarium voucher number 1625, conducted the taxonomic identification of this specimen. Chemical suppliers (Merck, Germany) provided all the analytical‐grade compounds that were employed in this study.

### 2.2 Microorganisms and Media

*S. mutans* (ATCC, 35668), *L. fermentum* (ATCC, 9338), *S. sanguinis* (ATCC, 1449), *S. salivarius* (ATCC, 1448), *S. aureus* (ATCC, 29737), *S. epidermidis* (ATCC, 49461), and *S. sobrinus* (ATCC, 27607) were among the microbial strains employed in this investigation (Table 1). Frozen bacteria were purchased from the Tehran Industrial Microorganism Collection Center (Iran). MRS agar, MRS broth, manitol salt agar, BHI broth, Muller‐Hinton agar (Ibresco, Iran), Mitis Salvarus agar (Qlab, Canada), and Vancomycin Antimicrobial Susceptibility discs (Sigma, USA) were among the culture mediums utilized.

**TABLE 1:** Independent variables used in this study.

Bacteria	Extract type	Concentration (ppm)
*Streptococcus mutans*		0.1
*Streptococcus sanguinis*	Ethanolic extract	1
*Streptococcus sobrinus*	Hydroethanolic extract	10
*Streptococcus salivarius*	Aqueous extract	100
*Staphylococcus aureus*		500
*Staphylococcus epidermidis*		1000
*Lactococcus fermentum*		

### 2.3 Preparation of Plant Extracts

To prevent the hydrolysis of the plants' bioactive compounds, the aerial part of the *T. polium* was dried for ten days at room temperature in the shade. The dry wood was then as thoroughly removed from the compounds as possible before it was ground into a fine powder. A mixture of 1000 mL of solvent (ethanol, ethanol‐water (50:50), and water) and about 350 g of powder were added, and the mixture was shaken at 37°C for 3–5 days (Table 1). After that, a vacuum pump was used to filter the mixture. The solvents were then extracted from the mixture's extract using a rotary vacuum evaporator. The Clevenger extraction method was used to finish the extraction procedure (Al‐Otaibi and AlMotwaa 2021). The different types and polarities of solvents have an impact on the rate at which secondary metabolites are extracted, which explains the variability in extract types. Accordingly, depending on the polarity of the immediate surroundings, the plant's effective bioactives enter the solvent phase and can exhibit a variety of inhibitory and lethal properties.

### 2.4 Gas Chromatography–Mass Spectroscopy (GC–MS) Analysis of Crude Extract

The obtained extract was put into a GC apparatus coupled to a mass spectrometer (GC–MS) model 5975 Agilent to determine the bioactive components (Table 2). The carrier gas, helium, had an ionization energy of 70 eV, a flow rate of 1 mL/min, and a mass spectrum range of 50 to 550 m/z. They were recognized through examination of the breakage patterns of the extract chemicals and comparison of the mass spectra from the proposed compounds with the spectrum described in the references (Farahbakhsh et al. 2021).

**TABLE 2:** Identified bioactive compounds in the extract of *Teucrium polium* L. by GC–MS.

Peak number	Retention time (min)	Compound name	Peak area (%)
1	7.1414	Artemisia triene	0.452
2	7.2534	2‐Pinene	0.243
3	7.5407	2,2‐dimethyl‐3‐methylene‐bicyclo [2.2.1]heptane	0.791
4	8.0762	2(10)‐Pinene	0.336
5	8.5241	2,5,5‐trimethyl‐3,6‐heptadien‐2‐ol	1.147
6	9.0743	2‐Oxabicyclo[2.2.2]octane, 1,3,3‐trimethyl—	9.889
7	9.5806	1,5‐Heptadien‐4‐one, 3,3,6‐trimethyl—	7.204
8	9.7218	3‐Decen‐5‐one	0.244
9	9.9652	1,5‐Heptadien‐4‐ol, 3,3,6‐trimethyl—	8.341
10	10.2525	1‐Hexanol, 6‐[(tetrahydro‐2H‐pyran‐2‐yl)oxy]—	0.411
11	10.3547	2H‐Pyran, 2‐[(1‐butyl‐2‐propynyl)oxy]tetrahydro—	0.227
12	10.9195	Bicyclo[3.1.1]heptan‐3‐ol, 6,6‐dimethyl‐2‐methylene‐, [1S‐(1.alpha.,3.alpha.,5.alpha.)]—	0.357
13	10.9925	(+)‐2‐Bornanone	27.726
14	11.2798	Pinocarvone	0.593
15	11.3577	(S)‐3,3,6‐Trimethylhepta‐1,5‐dien‐4‐yl acetate	4.349
16	11.7569	Terpineol	0.257
17	11.8007	(−)‐Myrtenol	0.889
18	13.0422	1,7‐Octadiene‐3,6‐diol, 2,6‐dimethyl—	0.396
19	13.0909	Bornyl acetate	0.456
20	14.7073	11‐Oxatetracyclo[5.3.2.0(2,7)0.0(2,8)]dodecan‐9‐one	0.319
21	14.941	(S)(+)‐Z‐13‐Methyl‐11‐pentadecen‐1‐ol acetate	0.228
22	15.126	Epoxy‐.alpha.‐terpenyl acetate	0.2
23	16.9225	1‐Adamantyl fluoroformate	0.534
24	17.0199	(−)‐5‐Oxatricyclo[8.2.0.0(4,6)]dodecane,12‐trimethyl‐9‐methylene‐, [1R‐(1R*,4R*,6R*,10S*)]—	0.905
25	17.3558	Resorcinol monoacetate	0.454
26	17.6334	10,10‐Dimethyl‐2,6‐dimethylenebicyclo[7.2.0]undecan‐5.beta.‐ol	2.231
27	17.7989	7.beta.‐(1‐hydroxy‐1‐methylethyl)‐4a.beta.‐methyl‐1a.beta.‐decahydrocyclopropa[d]naphthalene	0.227
28	18.0131	7’‐Oxaspiro[cyclopropane‐1,4′‐tricyclo[3.3.1.0(6,8)]nonan‐2′‐one]	0.519
29	18.1932	Bicyclo[3.1.1]heptan‐2‐one, 6,6‐dimethyl—	0.295
30	19.4493	1‐Imidazol‐1‐yl‐3‐methylbut‐2‐en‐1‐one	0.57
31	19.6538	Bicyclo[3.3.1]nonane‐2,7‐dione	0.181
32	19.907	R(−)3,7‐Dimethyl‐1,6‐octadiene	0.698
33	20.0628	Hexane, 1,6‐dibromo—	2.532
34	20.3403	But‐2‐enoic acid, amide, 3‐methyl‐N‐methallyl—	1.654
35	20.647	1H‐Imidazol‐2‐amine	7.896
36	21.056	Cyclohexane, eicosyl—	0.177
37	21.2702	Borinic acid, diethyl‐, 1‐cyclododecen‐1‐yl ester	0.194
38	22.0638	Carbonic acid, octadecyl 2,2,2‐trichloroethyl ester	0.212
39	22.3413	2‐Hexadecen‐1‐ol, 3,7,11,15‐tetramethyl‐, [R‐[R*,R*‐(E)]]—	0.639
40	23.1106	Azuleno[4,5‐b]furan‐2,7‐dione, 3,3a,4,5,9a,9b‐hexahydro‐3,6,9‐trimethyl‐, [3R‐(3.alpha.,3a.beta.,9a.beta.,9b.alpha.)]—	0.821
41	23.8603	(E)‐15,16‐Dinorlabda‐8(17),11‐dien‐13‐one	0.235
42	24.3472	5,5,8a‐Trimethyldecalin‐1‐one	0.656
43	25.5546	Eicosane	2.07
44	26.4212	Hexacosyl propyl ether	0.323
45	27.0639	1‐Octadecene	2.069
46	27.7845	Dodecahydropyrido[1,2‐b]isoquinolin‐6‐one	0.177
47	27.877	13‐Tetradecen‐1‐ol acetate	0.533
48	28.4904	Nonacos‐1‐ene	1.748
49	29.2305	Benzo[h]quinoline, 2,4‐dimethyl—	0.211
50	29.9169	N‐Methyl‐1‐adamantaneacetamide	0.509
51	31.5966	4‐tert‐Butylphenol, TMS derivative	0.607
52	32.2198	2‐Ethylacridine	2.2
53	36.1244	1‐Benzazirene‐1‐carboxylic acid, 2,2,5a‐trimethyl‐1a‐[3‐oxo‐1‐butenyl] perhydro‐, methyl ester	2.867

### 2.5 Antimicrobial Assays

#### 2.5.1 Disc Inhibition Zone Assay

The lyophilized bacteria were first cultivated in the appropriate culture medium. Streptococci were grown in a BHI broth medium in an incubator with CO_2_ at 37°C. *S. aureus* and *S. epidermidis* were grown in the BHI broth medium, whereas *L. fermentum* was grown in the MRS broth medium. The number of bacteria was adjusted to 10^8^ cfu/mL using the McFarland turbidity test after 24–48 h inoculation and cultured in a Petri dish containing agar culture medium; *S. aureus* and *S. epidermidis* in mannitol salt agar, Streptococci in Mitis salvarius agar, and *L. fermentum* in MRS agar. When the antibiotic‐empty disks were inoculated with various extract concentrations (0.1, 1, 10, 100, 500, and 1000 ppm), the bacteria‐containing culture media was transferred to the incubator at 37°C. The inhibitory zone that formed around them as a result of the extract's presence in the culture media was measured by the zone diameter after the 24‐h period (Sharifi‐Rad et al. 2022).

#### 2.5.2 Agar Well Inhibition Zone Assay

Using a Pasteur pipette, holes containing bacteria were produced within the plates, and 20 μL of extract from each dilution was added to the generated wells; the plates were placed in an incubator at 37°C for 24 h before being removed from the incubator. The zone diameter determined the surrounding inhibitory zone formed by the presence of the extract in the culture media (Hashemi et al. 2022).

#### 2.5.3 Minimum Inhibitory Concentrations (MIC) Assay

By using the McFarland turbidity test, the number of bacteria was adjusted to 10^8^ CFU/mL. The extract was then added to each well in 100 μL at various concentrations (0.1, 1, 10, 100, 500, and 1000 ppm), and the microbial suspension was added to the extracts. In place of the planned extract, the positive control wells (first row of the plate) contained 100 μL culture media, 20 μL bacterial suspension, and 100 μL physiological serum. The negative control wells (final row of the plate) contained only 100 μL of cultivating media, 20 μL of sterile physiological serum instead of bacterial suspension, and 100 μL of the intended extract. Before placing the microplates in the incubator, the optical density (OD) of the wells was measured with an ELISA reader; after 24 h, the OD of the wells was measured at a wavelength of 630 nm. There were six ∆OD numbers based on the concentrations used. Excel was used to create the regression equation of ∆OD using the formula ∆OD = a + bX, and by substituting zero for ∆OD in the obtained regression equation, X was equal to MIC (Shahraki‐Mojahed, Behzadmehr, and Beigomi 2021).

#### 2.5.4 Statistical Analysis

Table 1 shows the factors used in this investigation. SPSS (SPSS Inc., Chicago, IL, USA) software was used to compare data using one‐way analysis of variance (ANOVA) and Duncan's multiple range tests. All analyses were performed in triplicate, and differences in responses were examined using ANOVA at the *p* < 0.05 level.

## 3 Results and Discussion

### 3.1 Detection and Identification of Bioactive Compounds of Aqueous Extract of *T. polium*

**FIGURE 1:**
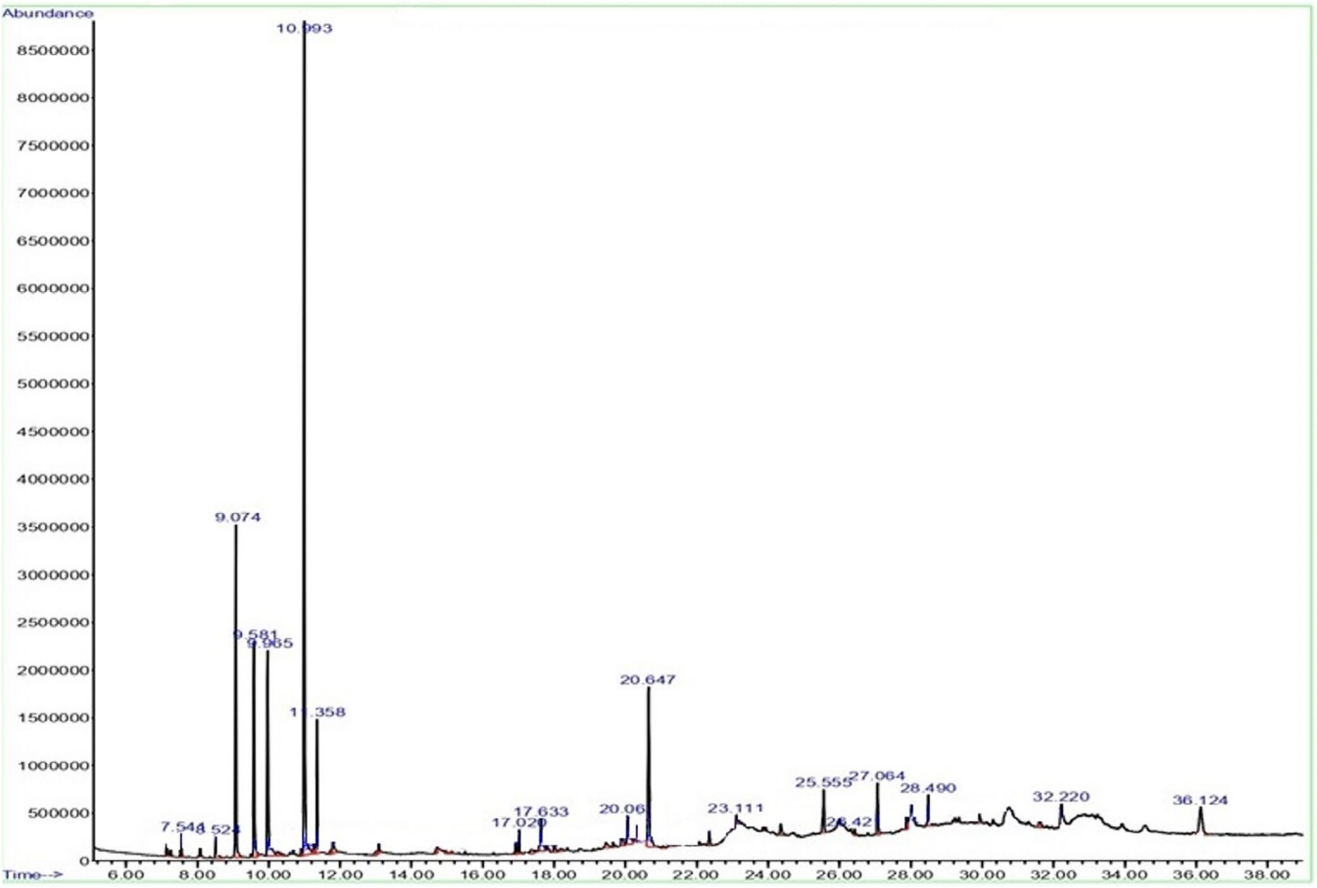
The GC–MS spectrum of the *Teucrium polium* L. extract.

The extract's GC profile is displayed in Figure 1. Fifty‐three primary and major bioactive compounds were found after looking at the retention times (RTs) of the GC spectra and the break patterns of the mass spectrum (Table 2). The relative percentage of each compound was also calculated using the area under the curve of each peak and comparing it to the overall area under the curve. Terpenes, fatty acids, polyphenols, flavonoids, coumarines, amino acids, alkaloids, and their derivatives dominated the several classes of phytochemical substances that were found in the extract. The most prevalent substances in the *T. polium* extract are 2‐Bornanone (27.73%), 2‐Oxabicyclo[2.2.2]octane,1,3,3‐trimethyl‐ (9.9%); 1,5‐Heptadien‐4‐ol, 3,3,6‐trimethyl‐ (8.34%); 1H‐Imidazol‐2‐amine (7.89%) and 1,5‐Heptadien‐4‐one, 3,3,6‐trimethyl‐ (7.20%) (Table 2). The results are consistent with prior research on the identification of plant components found in *T. polium*, such as α‐pinene, 1,8‐cineole, *cis*‐verbenol, caryophyllene oxide, β‐caryophyllene, and myrtenal (El Atki et al. 2020; Farahbakhsh et al. 2021; Sadrizadeh et al. 2018). The study investigated the chemical composition and antibacterial and antifungal activities of *T. polium* essential oil in Morocco. The oil was extracted through hydrodistillation and analyzed using gas chromatography and GC–MS. The *T. polium* essential oil with α‐Pinene being the major component (21.96%), Limonene (18.77%), and β‐Pinene (8.46%), exhibited strong antibacterial and antifungal activity. The oil's rich terpenes effectively combat microbial agents, with a broad spectrum of effects on molds, fungi, and bacteria (Chauiyakh et al. 2022).

### 3.2 Disc Diffusion Test

**FIGURE 2:**
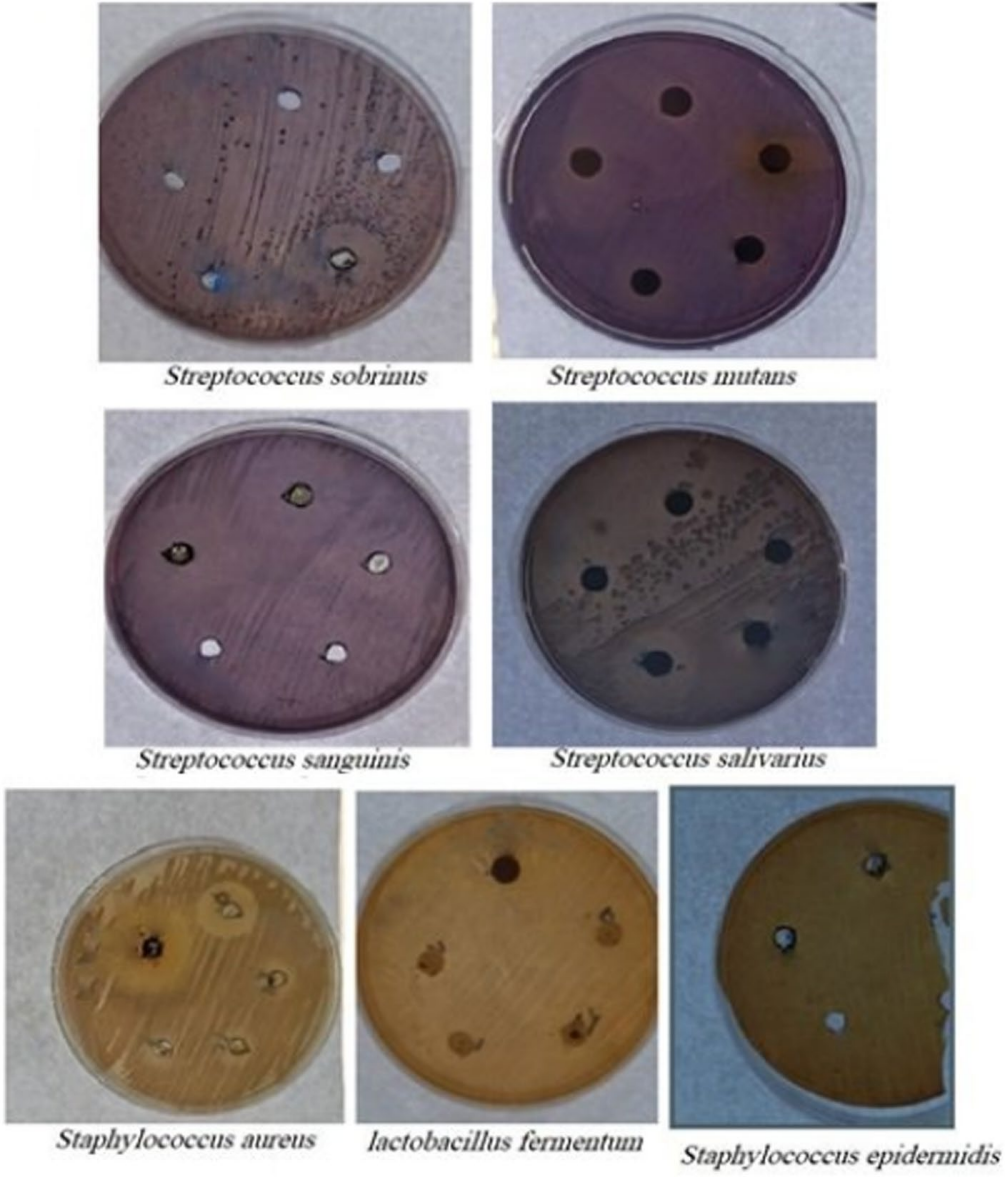
Antibacterial activity of *Teucrium polium* L. extract by the disk diffusion method.

Figure 2 and Tables 3, 4 show the antibacterial activity data of *T. polium* obtained using the disc diffusion method. The zone of inhibition (ZOI) index is significantly affected by the independent variables (type and concentration of extract and type of bacteria) as well as their interactions, according to the ANOVA results in Tables 3 and 4 (*p* < 0.0001). Although the clear zone sizes varied across the extract concentrations, all concentrations exhibited antibacterial action. Depending on the kind of solvent, every bacteria had a distinct ZOI diameter. Every extract had a concentration of 1 mg/mL (Table 3). The maximum ZOI diameter for *S. epidermidis* and *S. mutans* was 9.8 and 8.6 mm, respectively, and was related to the ethanolic extract. This was lower than the maximum ZOI diameter for erythromycin as a positive control (18 and 20 mm, respectively) and, of course, greater than other tested bacteria in all three extraction methods. However, for all of the studied microorganisms, ethanolic extracts outperformed aqueous and hydroethanolic extracts in terms of their considerable bactericidal impact. The ZOI diameter in *T. polium* was 11 mm, which is less than our findings and most likely a result of the extract's lower concentration (400 μg/mL) in comparison to ours (1000 μg/mL). According to a prior study by Yazdi and Behbahani (2013), the ZOI diameter in aqueous extract at a concentration of 40 mg/mL was 28 mm and in ethanolic extract at a concentration of 40 mg/mL was 57 mm. The essential oils (EOs) from *T. polium* were examined by Moghtader, Salari, and Farahmand (2013) for their ability to kill human pathogenic bacteria. Due to the high purity of the extracted EOs and their increased action, they found that the ZOI diameter of these EOs at a concentration of 1.5 μL/mL was 28 mm (Moghtader, Salari, and Farahmand 2013). According to the previous study the ZOI diameter was 10 mm for a 600 mg/mL methanolic extract concentration and 13 mm for a 400 mg/mL ethanolic extract concentration. Due to the high concentration of bioactive substances, including terpenes, flavonoids, and polyphenols, *T. polium* extract has been demonstrated to have inhibitory effects against oral bacteria. According to reports, these substances possess antibacterial capabilities that can damage the cell membrane of bacteria and prevent their growth and reproduction (Moghtader, Salari, and Farahmand 2013).

**TABLE 3:** The comparison of the interaction effect of bacterial type and extract type by disc diffusion, well diffusion, and minimum inhibitory concentration (MIC) methods.

Extraction type	Bacteria type	Disc inhibition zone (cm) (*n* = 18)	Well inhibition zone (cm) (*n* = 18)	MIC (PPM) (*n* = 3)
Ethanolic	*Streptococcus mutans*	$0.86\pm 0.23^{\mathrm{def}}$	$0.86\pm 0.22^{\mathrm{ab}}$	$1614.25\pm 5.91^{h}$
	*Streptococcus sanguinis*	$0.55\pm 0.19^{\mathrm{hj}}$	$0.48\pm 0.16^{\mathrm{be}}$	$1545.70\pm 7.10^{i}$
	*Streptococcus sobrinus*	$0.40\pm 0.14^{\mathrm{hj}}$	$0.40\pm 0.14^{\mathrm{cf}}$	$1516.20\pm 6.92^{j}$
	*Streptococcus salivarius*	$0.58\pm 0.20^{\mathrm{ej}}$	$0.58\pm 0.20^{\mathrm{bd}}$	$1879.30\pm 8.77^{g}$
	*Staphylococcus aureus*	$0.55\pm 0.14^{\mathrm{hj}}$	$0.85\pm 0.12^{\mathrm{ac}}$	$1903.40\pm 9.98^{g}$
	*Staphylococcus epidermidis*	$0.98\pm 0.19^{\mathrm{de}}$	$1.03\pm 0.21^{a}$	$2373.30\pm 10.10^{c}$
	*Lactococcus fermentum*	$0.58\pm 0.20^{\mathrm{ej}}$	$0.55\pm 0.19^{\mathrm{be}}$	$1360.70\pm 5.31^{l}$
Hydroethanolic	*Streptococcus mutans*	$0.33\pm 0.18^{\mathrm{hj}}$	$0.25\pm 0.09^{\mathrm{def}}$	$1243.80\pm 8.94^{m}$
	*Streptococcus sanguinis*	$0.46\pm 0.16^{\mathrm{hj}}$	$0.41\pm 0.14^{\mathrm{bf}}$	$2442.30\pm 7.50^{b}$
	*Streptococcus sobrinus*	$0.43\pm 0.15^{\mathrm{hj}}$	$0.40\pm 0.14^{\mathrm{cf}}$	$1896.00\pm 9.23^{g}$
	*Streptococcus salivarius*	$0.35\pm 0.12^{\mathrm{hj}}$	$0.41\pm 0.14^{\mathrm{bf}}$	$1523.40\pm 7.62^{\mathrm{ij}}$
	*Staphylococcus aureus*	$0.20\pm 0.10^{\mathrm{hj}}$	$0.20\pm 0.10^{\mathrm{def}}$	$1959.00\pm 9.81^{f}$
	*Staphylococcus epidermidis*	$0.56\pm 0.16^{\mathrm{hj}}$	$0.63\pm 0.18^{\mathrm{ad}}$	$2201.00\pm 9.23^{d}$
	*Lactococcus fermentum*	$0.16\pm 0.09^{\mathrm{ij}}$	$0.18\pm 0.10^{\mathrm{def}}$	$1416.75\pm 5.36^{k}$
Aqueous	*Streptococcus mutans*	−	−	$1506.10\pm 6.46^{j}$
	*Streptococcus sanguinis*	$0.16\pm 0.09^{\mathrm{ij}}$	$0.16\pm 0.09^{\mathrm{def}}$	$2425.00\pm 13.27^{b}$
	*Streptococcus sobrinus*	−	−	$2598.60\pm 12.93^{a}$
	*Streptococcus salivarius*	$0.13\pm 0.07^{\mathrm{ij}}$	$0.31\pm 0.10^{\mathrm{def}}$	$2393.30\pm 9.93^{c}$
	*Staphylococcus aureus*	$0.16\pm 0.09^{\mathrm{ij}}$	$0.25\pm 0.09^{\mathrm{def}}$	$2056.80\pm 9.35^{e}$
	*Staphylococcus epidermidis*	$0.08\pm 0.04^{\mathrm{ij}}$	$0.10\pm 0.05^{\mathrm{ef}}$	$2035.20\pm 10.16^{e}$
	*Lactococcus fermentum*	−	$0.23\pm 0.12^{\mathrm{def}}$	$2036.20\pm 8.42^{e}$
Erythromycin (control +)	*Streptococcus mutans*	$2.00\pm 0.01^{\mathrm{bc}}$	−	−
	*Streptococcus sanguinis*	$1.40\pm 0.02^{\mathrm{cd}}$	−	−
	*Streptococcus sobrinus*	$2.00\pm 0.02^{\mathrm{bc}}$	−	−
	*Streptococcus salivarius*	$2.50\pm 0.05^{b}$	−	−
	*Staphylococcus aureus*	$3.50\pm 0.04^{a}$	−	−
	*Staphylococcus epidermidis*	$1.80\pm 0.02^{\mathrm{bc}}$	−	−
	*Lactococcus fermentum*	$1.00\pm 0.01^{\mathrm{de}}$	−	−
Distilled water (control −)	*Streptococcus mutans*	−	−	−
	*Streptococcus sanguinis*	−	−	−
	*Streptococcus sobrinus*	−	−	−
	*Streptococcus salivarius*	−	−	−
	*Staphylococcus aureus*	−	−	−
	*Staphylococcus epidermidis*	−	−	−
	*Lactococcus fermentum*	−	−	−

*Note:* Means ± SE with different letters in each column type indicate significant difference (*p* < 0.05). For the disc and well diffusion tests, the concentration of the plant extract applied was 1000 ppm.

**TABLE 4:** The comparison of the interaction effect between bacterial types and extract concentration in different extracts by disc diffusion and well diffusion methods.

Tract concentration (PPM)	Ethanolic extract		Hydroethanolic extract		Aqueous extract		Erythromycin inhibition zone (cm)
	Disc inhibition zone (cm)	Well inhibition zone (cm)	Disc inhibition zone (cm)	Well inhibition zone (cm)	Disc inhibition zone (cm) (*n* = 3)	Well inhibition zone (cm)	
*Streptococcus mutans*							
10	−	−	−	−	−	−	
100	$1.20\pm 0.02^{g}$	$1.20\pm 0.02^{i}$	−	−	−	−	$2\pm 0.01^{c}$
500	$1.50\pm 0.04^{d}$	$1.70\pm 0.03^{d}$	−	$0.50\pm 0.01^{g}$	−	−	
1000	$2.50\pm 0.01^{a}$	$2.30\pm 0.04^{a}$	$2.00\pm 0.02^{a}$	$1.00\pm 0.03^{f}$	−	−	
*Streptococcus sanguinis*							
100	−	−	−	−	−	−	
500	$\;1.30\pm 0.01^{f}$	$1.20\pm 0.05^{i}$	$1.10\pm 0.05^{f}$	$1.00\pm 0.04^{f}$	−	−	$1.40\pm 0.02^{e}$
1000	$2.00\pm 0.05^{b}$	$1.70\pm 0.01^{d}$	$1.70\pm 0.02^{b}$	$\;1.50\pm 0.05^{b}$	$1.00\pm 0.03^{a}$	$1.00\pm 0.02^{b}$	
*Streptococcus sobrinus*							
100	−	−	−	−	−	−	
500	$1.00\pm 0.01^{h}$	$1.00\pm 0.03^{j}$	$1.00\pm 0.01^{g}$	$1.00\pm 0.01^{f}$	−	−	$2.00\pm 0.02^{c}$
1000	$1.40\pm 0.05^{e}$	$1.40\pm 0.02^{g}$	$1.60\pm 0.02^{c}$	$1.40\pm 0.02^{c}$	−	−	
*Streptococcus salivarius*							
100	−	−	−	−	−	−	
500	$1.50\pm 0.05^{d}$	$1.50\pm 0.04^{f}$	$\;0.80\pm 0.02^{h}$	$1.00\pm 0.05^{f}$	−	$0.9\pm 0.05^{d}$	$2.50\pm 0.05^{b}$
1000	$2.00\pm 0.03^{b}$	$2.00\pm 0.02^{c}$	$1.30\pm 0.02^{d}$	$1.50\pm 0.02^{b}$	$\;0.80\pm 0.02^{b}$	$1.00\pm 0.02^{b}$	
*Staphylococcus aureus*							
1	−	−	−	−			
10	−	$\;0.80\pm 0.02^{l}$	−	−	−	−	
100	$0.80\pm 0.02^{i}$	$0.90\pm 0.01^{k}$	−	−	−	−	
500	$1.00\pm 0.03^{h}$	$1.30\pm 0.02^{h}$	−	−	−	$0.50\pm 0.00^{f}$	$3.50\pm 0.04^{a}$
1000	$1.50\pm 0.01^{d}$	$1.60\pm 0.02^{e}$	$1.20\pm 0.03^{e}$	$1.20\pm 0.03^{d}$	$1.00\pm 0.01^{a}$	$1.00\pm 0.01^{b}$	
*Staphylococcus epidermidis*							
1	−	−	−	−	−	−	
10	$0.60\pm 0.01^{j}$	$\;0.60\pm 0.01^{m}$					
100	$1.50\pm 0.01^{d}$	$1.60\pm 0.02^{e}$	$0.50\pm 0.01^{i}$	$0.50\pm 0.01^{g}$	−	−	
500	$1.80\pm 0.04^{c}$	$1.93\pm 0.04^{c}$	$1.20\pm 0.05^{e}$	$1.40\pm 0.02^{c}$	−	−	$1.80\pm 0.02^{d}$
1000	$2.00\pm 0.00^{b}$	$2.10\pm 0.05^{b}$	$1.70\pm 0.01^{b}$	$1.90\pm 0.02^{a}$	$0.50\pm 0.01^{c}$	$0.60\pm 0.01^{e}$	
*Lactococcus fermentum*							
100	−	−	−	−	−	−	
500	$1.50\pm 0.02^{d}$	$1.30\pm 0.02^{h}$	−	−	−	−	$1.00\pm 0.01^{f}$
1000	$2.00\pm 0.03^{b}$	$2.00\pm 0.05^{c}$	$1.00\pm 0.01^{g}$	$1.13\pm 0.07^{e}$	−	$1.40\pm 0.04^{a}$	

*Note:* Means ± SE (*n* = 3) with different letters in each column type indicate significant difference (*p* < 0.05).

### 3.3 Agar Well Diffusion Test

Tables 3 and 4 reveal that the effect of independent factors (type and concentration of extract and type of bacteria) and their interactions on the growth index of examined bacteria was significant (*p* < 0.0001). Table 4 shows the clear zone diameters of several extracts at varying doses. The extracts have a broad antibacterial spectrum against the studied microorganisms, with inhibition zone diameters ranging from 1 to 10.3 mm. These findings were consistent with the findings of Chedia et al. (2013), who used the well diffusion method to investigate the antibacterial activity of *T. polium* extract against *S. aureus* and *Escherichia coli* . There was a significant difference in the inhibitory effect of ethanolic extract for all types of tested bacteria (*p* < 0.05), with the highest inhibition zone for *S. epidermidis* , *S. aureus* , and *S. mutans* , whereas the hydroethanolic and aqueous extraction methods for all bacteria showed the same inhibition zone statistically (*p* > 0.05), but it was much lower than the ethanolic extraction method. The ZOI diameter is insufficient to compare the effects of antimicrobial compounds on two bacteria and cannot identify whether the antibacterial property of the substance is greater or lesser. Rather, it is just a qualitative tool for determining whether or not the required microorganism has antibacterial capabilities. The more accurate method of evaluating light absorption in different concentrations is used to compare the effect of antibacterial agents. In reality, the MIC test is complemented by these two assays (disc diffusion and agar well diffusion). Several research have been conducted to examine the antibacterial impact of *T. polium* aqueous, ethanolic, methanolic, and EOs on a variety of bacteria (Ben Othman et al. 2017; Chedia et al. 2013; El Atki et al. 2020; Keykavousi et al. 2016; Nacéra et al. 2013). Because the bacteria in these tests were not the same as in our investigations, the results were not presented in detail, but the results demonstrate that *T. polium* has a very good antibacterial impact on a wide range of bacteria, which is consistent with the results of our study. The only study that contradicted our findings was that of Saleh et al. (2020), who concluded that EOs of *T. polium* with MICs ranging from 62.5 to 125 g/mL have poor antibacterial efficacy. The quantity of hydroxyl groups in these chemicals is said to be closely related to their harmful effect on microorganisms. Tannin is another antibacterial component found in the *T. polium* plant. These chemicals have antibacterial actions by reducing microbe resistance and inhibiting microbial enzyme activity (Seabra et al. 2006). Furthermore, Wong and Kitts (2006) discovered that the antimicrobial properties of some medicinal plants are most likely due to phenolic compounds with a polar isopropyl functional group, which can potentially disrupt cell function and membrane integrity due to the presence of hydrophobic properties. Along with ascorbic acid, phosphorylated chemicals, and proteins, phenolic compounds are natural chelating agents in fresh foods. Also, as a ligand, phenolic compounds can bind to iron metal and chelate it. Transition metal–ligand complexes, such as iron and copper, inhibit bioavailability for bacterial growth (Wong and Kitts 2006). The study explores *T. polium* extract's antibacterial properties, including bioactive compounds such as terpenes, flavonoids, polyphenols, and tannins. Phenolic compounds disrupt cell function and membrane integrity, acting as natural chelating agents and inhibiting bacterial growth.

### 3.4 MIC Results

Tables 3 and 4 demonstrate that the effect of independent factors (type and concentration of extract and type of bacteria) and their interactions on the MIC was significant (*p* < 0.0001). Based on the results, all *T. polium* samples had MIC values ranging from 1243 to 2443 ppm against all Gram‐positive bacteria tested. The most resistant and sensitive type of bacteria to each type of extract were identified in our investigation, which was not accessible in a comparable study in terms of the type of bacteria for comparison. The concentration of the extract in which the bacteria's growth was inhibited differed depending on the type of solvent in the case of all microorganisms. The development of *S. mutans* was suppressed by hydroethanolic extract at a concentration of 1.24 mg/mL, aqueous extracts at 1.5 mg/mL, and ethanolic extract of *T. polium* plant at 1.6 mg/mL. In fact, the hydroethanolic extract inhibited the development of *S. mutans* bacteria more effectively.

Sesquiterpenes, notably oxygenated molecules such as 6‐epi‐shyobunol, t‐muurolol, and germacrene D‐4‐ol, have been linked to the antibacterial action of *T. polium* EOs. The antibacterial properties of *T. decussatus* could be related to the extract's main components, such as carvacrol and p‐cymene. Several investigations have found that carvacrol and p‐cymene have antibacterial action (Saleh et al. 2020). The previous study discovered that Algerian *T. polium* has modest antibacterial activity, with MIC values ranging from 3 to 5 μL/mL (Nacéra et al. 2013).

The quantity of saliva *S. mutans* colonies decreased after consuming gum containing the aqueous extract of this plant, according to Khoramian Tusi et al. (2018). In another investigation, Khoramian Tusi found that drinking 2 g/L mouthwash with *T. polium* aqueous extract for 2 weeks reduced the number of salivary *S. mutans* colonies considerably. These research’ findings were congruent with ours. They also claimed that phenolic chemicals found in the *T. polium* plant can diminish the quantity of salivary *S. mutans* . Bravo (1998) demonstrated that Gram‐positive and Gram‐negative bacteria are susceptible to phenolic chemicals, which are found in many plants. *T. polium* hydroethanolic and aqueous extracts inhibited the development of *S. sanguinis* at concentrations of 1.54 mg/mL of methanolic and ethanolic extracts, 1.7 mg/mL of hydromethanolic extract, and 2.4 mg/mL of hydroethanolic and aqueous extracts. In fact, methanolic and ethanolic extract inhibits the growth of *S. sanguinis* bacteria more effectively. *S. epidermidis* bacterial growth was inhibited by *T. polium* plant extracts at concentrations of 2 mg/mL from the aqueous extract, 2.2 mg/mL from the hydroethanolic extract, and 2.4 mg/mL from the ethanolic extract. In fact, the aqueous extract was more effective at preventing *S. epidermidis* bacterial growth. A concentration of 1.36 mg/mL of ethanolic extract, 1.4 mg/mL of hydroethanolic extract, and 2.03 mg/mL of aqueous extract *T. polium* all suppressed the growth of *L. fermentum* bacteria. In fact, *L. fermentum* bacterium growth was more effectively inhibited by the plant's ethanolic extract. No comparable study existed for review and comparison. The variance in bacterial MIC of *T. polium* extract in different research may be due to differences in harvesting time, technique of extraction, storage conditions of the culture medium in the laboratory, and strains utilized in the laboratory. Furthermore, different varieties of *T. polium* plant may have different antibacterial chemical compounds. Holetz et al. (2002) presented the values of the MIC in research on plant extracts in such a way that the MIC of bacterial growth in amounts of 100 mg/mL indicates that the extract has high antibacterial action between 100 and 500 mg/mL. Values between 500 and 1000 mg/mL indicate modest antimicrobial activity, whereas values greater than 1000 mg/mL indicate the absence of antibacterial activity in the studied extract. As a result, the antibacterial action of *T. polium* plant is established in a fair degree in this investigation. According to Khoramian Tusi et al. (2018), the antibacterial activities of *T. polium* bioactive compounds are dependent on the amount and position of phenolic hydroxyl groups. The study explores *T. polium* extract's antibacterial activity and potential mechanisms of action, supporting previous research as a natural alternative to traditional antibiotics. Variations in MIC may be due to harvesting time, extraction techniques, storage conditions, and laboratory strains.

## 4 Conclusion

The extracts of *Teucrium polium* L. demonstrated significant antimicrobial activity against oral microorganisms responsible for dental caries. The results from disc and well diffusion experiments indicated that the ethanolic extract exhibited the strongest effects, followed closely by the hydroethanolic extract, while aqueous solvents were less effective. Given these findings, it is advisable to use the antibacterial extracts of this plant with the most effective solvent tailored to target specific bacteria. Notably, both ethanolic and hydroethanolic extracts at a concentration of 2.44 mg/L inhibited all bacteria examined. Therefore, we recommend the development of mouthwashes containing these extracts to effectively prevent tooth decay. For future applications, further research should explore the formulation of mouthwashes that maintain the efficacy of these extracts while ensuring safety for daily use. Additionally, if the use of alcoholic extracts is a concern, an aqueous extract at a concentration of 2.6 mg/L could be considered an alternative. Future studies should also focus on isolating and characterizing the active compounds within *T. polium* to elucidate their mechanisms of action against oral pathogens. Moreover, research involving bacteria cultured from the saliva of individuals with high caries susceptibility would provide valuable insights into the practical applications of these extracts. Comparative studies should be conducted to evaluate the efficacy of *T. polium* extracts against conventional treatments, such as antibiotics, chlorhexidine, and fluoride mouthwashes. These investigations could pave the way for innovative natural therapies in dental care, ultimately contributing to better oral health outcomes.

## Author Contributions

**Kamand Javadpour:** conceptualization (equal), data curation (equal), formal analysis (equal), funding acquisition (equal), investigation (equal), methodology (equal), project administration (equal), resources (equal), software (equal), validation (equal). **Hajar Shekarchizadeh:** resources (equal), software (equal), supervision (equal), validation (equal), visualization (equal), writing – original draft (equal), writing – review and editing (equal). **Mohammad Goli:** conceptualization (equal), data curation (equal), formal analysis (equal), funding acquisition (equal), investigation (equal), methodology (equal), project administration (equal), resources (equal), software (equal), supervision (equal), validation (equal), visualization (equal), writing – original draft (equal), writing – review and editing (equal). **Helena Moradiyan Tehrani:** investigation (equal), methodology (equal), writing – original draft (equal), writing – review and editing (equal).

## Conflicts of Interest

The authors declare no conflicts of interest.

## Data Availability

The data that support the findings of this study are available from the corresponding author upon reasonable request.
