# Correction to “Antimicrobial Efficacy of *Teucrium polium* L. Extracts for Dental Caries: Green Extraction Techniques and Bioactive Compounds”

**DOI:** 10.1002/fsn3.70087

**Published:** 2025-03-18

Correction to “Antimicrobial Efficacy of *Teucrium polium* L. Extracts for Dental Caries: Green Extraction Techniques and Bioactive Compounds” Published in *Food Science & Nutrition*, 2025; 13:e4718. https://doi.org/10.1002/fsn3.4718

The affiliations of authors Mohammad Goli and Helena Moradiyan Tehrani are revised as follows:

Department of Food Science and Technology, Laser and Biophotonics in Biotechnologies Research Center, Isfahan (Khorasgan) Branch, Islamic Azad University, Isfahan, Iran.

Department of Food Science and Technology, Damghan Branch, Islamic Azad University, Semnan, Iran.

We apologize for this error.

